# Recovery of Interdependent Networks

**DOI:** 10.1038/srep22834

**Published:** 2016-03-09

**Authors:** M. A. Di Muro, C. E. La Rocca, H. E. Stanley, S. Havlin, L. A. Braunstein

**Affiliations:** 1 Instituto de Investigaciones Físicas de Mar del Plata (IFIMAR)-Departamento de Física, Facultad de Ciencias Exactas y Naturales, Universidad Nacional de Mar del Plata-CONICET, Funes 3350 (7600) Mar del Plata, Argentina; 2 Center for Polymer Studies, Boston University, Boston, Massachusetts 02215, USA; 3 Department of Physics, Bar Ilan University, Ramat Gan, Israel

## Abstract

Recent network research has focused on the cascading failures in a system of interdependent networks and the necessary preconditions for system collapse. An important question that has not been addressed is how to repair a failing system before it suffers total breakdown. Here we introduce a recovery strategy for nodes and develop an analytic and numerical framework for studying the concurrent failure and recovery of a system of interdependent networks based on an efficient and practically reasonable strategy. Our strategy consists of repairing a fraction of failed nodes, with probability of recovery γ, that are neighbors of the largest connected component of each constituent network. We find that, for a given initial failure of a fraction 1 − *p* of nodes, there is a critical probability of recovery above which the cascade is halted and the system fully restores to its initial state and below which the system abruptly collapses. As a consequence we find in the plane γ − *p* of the phase diagram three distinct phases. A phase in which the system never collapses without being restored, another phase in which the recovery strategy avoids the breakdown, and a phase in which even the repairing process cannot prevent system collapse.

In recent years researchers have attempted to understand the topological structure and self-organization of complex systems. The field of complex networks, which characterizes components of a complex system as nodes and their interactions as links, has emerged as a natural outgrowth of this quest. Studies of the Internet, human and animal societies, climate systems, physiological systems, transportation systems, biochemical reactions, and food webs in ecosystems are only few examples of systems that are better understood using complex network theory^1^,^2^,^3^,^4^,^5^,^6^,^7^,^8^,^9^,^10^,^11^,^12^,^13^,^14^,^15^,^16^,^17^,^18^,^19^. However, it was recently demonstrated that many complex systems cannot be described adequately as single isolated networks but should be represented as interdependent networks, which are characterized by connectivity links within each network and dependency links between networks^20^,^21^. Technological infrastructures provide the most obvious examples. Electrical, gas, and water networks rely on telecommunications networks for their control systems. Water systems are used to cool generators in an electrical system. Nearly every infrastructure network depends on the power grid to function. Such macro systems are much more complex and vulnerable compared to isolated networks. For interdependent networks the distinction between *internal* connectivity links within each network and *interdependent* links between the networks represents new challenges and the interest and research in these multiple coupled systems has recently rapidly expanded. In September 2003 a tree fell on a transmission line in Switzerland and triggered a cascade of failures that left 53 million people in the dark, most of them in Italy. This additional massive blackout to the growing list of global large-scale catastrophic events has motivated the study of robustness and cascading failures in interdependent networks. Using percolation theory, Buldyrev *et al.*^20^ developed a framework for studying interdependent networks and found that the coupled system behaves very different from a single isolated network and is significantly more fragile. In contrast to the percolation of single networks where the transition is in general continuous, an abrupt first-order percolation transition was found in interdependent networks where near the critical point a tiny fraction of node failures can cause cascading failure and system collapse.

It was also found^22^ that reducing interdependencies between networks below a critical value yields a continuous percolation transition. Very recently, Gao *et al.*^23^, Schneider *et al.*^24^ and Valdez *et al.*^25^ showed that backing up high-degree interdependent nodes enhances the robustness of a coupled system. It was also found that networks with assortative dependency (i.e., nodes with similar degrees in both networks tend to be dependent) are more robust than networks with random dependency^26^,^27^,^28^. Previous resilience studies have focused on failure propagation and the breakdown of systems of coupled networks. Much work has been devoted to the design of control and mitigation strategies^22^,^26^,^27^,^28^ to avoid catastrophic events and to heal failures as they occur. In order to reduce overload failures in power systems, some proposed control strategies consist of simply strengthening the capacity or reducing the load of groups of nodes. Mitigation can also be achieved by “islanding” nodes, i.e., separating certain clusters from the main power grid and powering them with independent alternative sources as solar or wind power^29^. Nevertheless, in real-world scenarios nodes can be repaired or recovered. Complex networks with heterogeneous distribution of loads may undergo a global cascade of overload failures when highly loaded nodes or edges are removed due to attacks or failures. Since a small attack or failure has the potential to trigger a global cascade, a fundamental question of much interest is regarding the possible strategies of defense to prevent the cascade from propagating through the entire network. Motter^30^ introduced a strategy of defense to prevent a global cascade of overload failures in isolated heterogeneous networks using a selective removal of nodes and edges right after the initial attack or failure. This intentional removal of network nodes and edges drastically reduces the size of the cascade. Majdandzic *et al.*^31^ studied a failure recovery model in isolated networks where the failures are due to lack of support within the networks. In^31^ after an inactive period of time a significant part of the damaged network is capable, due to internal fluctuations, of spontaneously becoming active again. However, repairing interdependent networks that experience a cascade of failures is a possibility that has not yet been taken into consideration.

In this work, we develop a model for the competition between the cascading failures and the restoration strategy that repairs failed nodes in the boundary of the functional network and reconnects them to it (see Fig. 1). The reasoning behind this repairing strategy is based on the fact that (a) in many real systems it is easier to repair boundary nodes (for example, in a transportation system one needs to bring equipment to the damaged site and it is easier to bring it near using the existing transportation system) and (b) fixing a node that is not in the boundary will cause the node to fail in the next step since it is not connected to the giant component and thus such a repair will be a wasted effort. In order to determine the recovery probability necessary to protect a system from collapse, we develop a theoretical model that is solved using random percolation theory. We present numerical solutions for the evolution of the theoretical process as well as for the steady states and compare them with simulations. We find that there is a critical probability *γ*_*c*_ that depends on *p* that separates a regime of full system fragmentation from a regime of complete system restoration.

## Results

### Model

For the sake of simplicity and without loss of generality, we consider two interdependent networks *A* and *B*.

#### Stochastic Model

Both networks have the same number of nodes *N*. Within each network the nodes are randomly connected through connectivity links with a degree distribution *P*^*i*^(*k*), where *i* = *A* or *B*. Pairs of nodes across the two networks are randomly connected one-to-one via bidirectional interdependent links as in Buldyrev *et al.*^20^.

**Figure 1:**
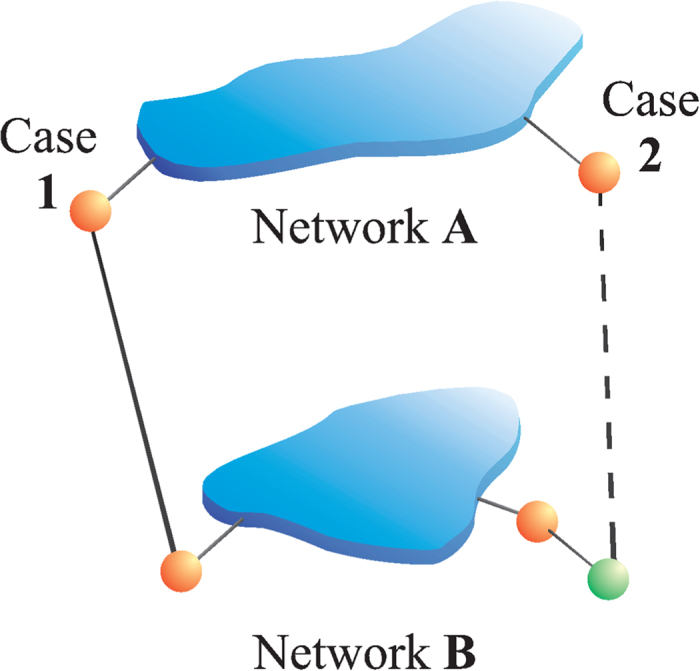
Schematic rules of the failure-recovery strategy. The GCs of networks *A* and *B* are shown (blue). In orange we mark boundary nodes at a distance 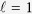 from their respectively GCs and in green a node with a distance 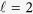 from the GC in B. Case 1: Two interconnected failed nodes at a distance 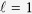 from their respectively GCs are repaired with probability *γ*. Case 2: If at least one of the two interconnected failed nodes is at a distance 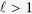 from its GC, we do not recover these nodes. Note that this type of recovery is practical and realistic, since in real infrastructure it is usually more convenient to repair boundary nodes which are next to the functional infrastructure GC.

We assume that at the initial stage a fraction 1 − *p* of nodes in network *A* fail. The failure spreads in network *A* through connectivity links and all the nodes that do not belong to the functional giant component (GC) of network *A* fail and it is assumed that they become dysfunctional. The failed nodes in network *A* no longer support their corresponding nodes in network *B* through their interdependent links, and those nodes in network *B* that were dependent on the failed nodes in *A* also fail. If the fraction of the initially-failed nodes in *A* is above 1 − *p*_*c*_, where *p*_*c*_ is the critical threshold, and there is no repair strategy, a catastrophic cascade of failures occurs and the system abruptly collapses. Our model assumes a process of recovery that is immediately applied at the first step of the cascade of failures with the objective of avoiding or delaying the collapse of the system. In this process certain failed nodes are recovered according to the following rules:

1. If a failed node in one network is at a distance 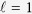 from its GC (we denote the collection of nodes at distance 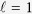 from the GC as the boundary of the GC) and has an interdependent link with a failed node in the other network that is also at a distance 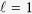 from its corresponding GC, this pair of nodes belongs to the mutual boundary and the two are repaired with a probability γ.
2. If the interdependent node in the other network does not belong to the boundary, none of them is repaired. Figure 1 sketches the recovery strategy.

It is important to clarify that when a node of the boundary is restored, not only all its connections with the GC are reactivated, but also its connectivity links with other restored nodes from the same network are recovered (if they were connected originally).

We denote by *n* = 0, 1, …. the time steps of the cascading process. In the simulations at *n* = 0 a fraction 1 − *p* of nodes fails in network *A*. From the fraction *p* of nodes that survive, only those within the GC are regarded as functional while the others are dysfunctional and considered as failed nodes. After the initial failure the damage in *A* propagates to network *B* through the interdependent links, as the conventional process of cascading failures introduced in ref. 20, but before spreading the failures back to network *A* we restore the interdependent nodes that belong to the mutual boundary of both networks *A* and *B* with a probability γ. The rules of the model for any stage *n* are given by:

- Stage *n* in *A*
  1. Functional nodes fail if they lose support from their counterpart nodes in *B* at stage *n* − 1.
  2. From the survivors, those nodes that belong to the GC of *A* remain functional while the others fail.
- Stage *n* in *B*:
  1. Functional nodes become dysfunctional if they lose support from network *A* due to the cascade of failures at stage *n*.
  2. The remaining nodes fail if they do not belong to the GC of *B*.
  3. Interdependent nodes in the mutual boundary of the GCs of networks *A* and *B* are restored with probability *γ*. All their connections with the respective GC are reactivated and also the links between restored boundary nodes, if they were connected before the failure.

This procedure is repeated until a steady state is reached, which depends upon *γ* and *p*. In this state there are no finite clusters in any network and the fraction of nodes that belongs to the GC, 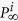, *i* = *A*, *B* in both networks, is the same because any node in each network is supported through interdependent links by the other node in the other GC.

#### Theoretical Approach

In order to solve our theoretical model, we use the generating function formalism^32^,^33^ extended to interdependent networks^20^,^23^,^25^,^28^,^34^,^35^, which is based on two generating functions in which 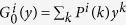 is the generating function of the degree distribution, 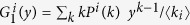 is the generating function of the excess degree distribution, and 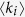 is the average degree of the network, with *i* = *A*, *B*. Using this formalism, we denote by *g*_*A*_[*x*] (*g*_*B*_[*y*]) the order parameter 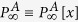 (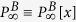) evaluated at *x* (*y*), then for the network *A*


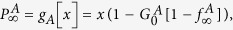


where 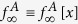 satisfies the self-consistent equation


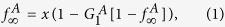


where 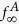 is the probability that an infinite branch expands the system in network *A*^23^,^25^,^28^,^33^,^34^,^35^. The same equations and definitions hold for network *B* with


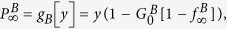


and


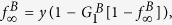


where also 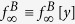.

As our theory is based on node percolation where finite clusters are not regarded as functional, dysfunctional nodes are failed nodes and nodes that belong to finite clusters are also failed. We denote by 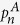 (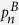) the effective fraction of nodes remaining in network *A* (*B*) after the cascade of failures and before repairing at step *n*. At stage *n* = 0 we have a fraction 1 − *p* of nodes from network *A* that fail and therefore 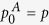 and 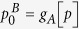 (for a detailed description of the process see Supplementary Information*: Theory*). After the initial cascade that goes from network *A* to network *B*, the process of recovery begins. At stage *n* the fraction of nodes in the GC of networks *A* and *B* is given by


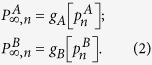


The nodes that are repaired are those that belong to the mutual boundary of both GCs. The fraction of nodes that are in the boundary of each GC can be written as


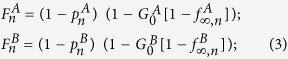


where the factor 
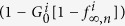
 is the probability that a node is connected to the GC at stage *n* and the factor 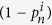 is the fraction of nodes that fail, with *i* = *A*, *B*. The mutual boundary, which is the fraction of nodes in the boundary of network *B* that are interconnected via dependency links to the nodes in the boundary of network *A* at stage *n*, can be written as


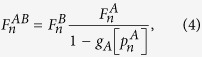


where 
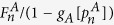
 is the conditional probability that a node belongs to the boundary of the GC of network *A* given that it is interconnected via an interdependent link with a node that belongs to the boundary of the GC of network *B*.

Next we compute the fraction of nodes in the GCs, 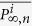, after repairing at stage *n* by adding a fraction *γ* of the mutual boundary to the values of Eq. (2)


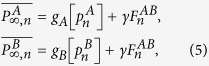


where the bar indicates the relative size of the order parameter of the enlarged GCs due to the restoration process.

Finally, we compute the fraction of remaining nodes in each network after the recovery process 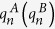 by solving the pair of transcendental equations


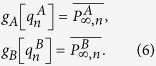


Then for any stage *n* > 0 the fraction of nodes remaining after the cascade of failures and before the repairing process in each network is given by


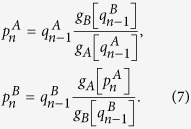


The process is iterated until the steady state is reached, when there are no more nodes belonging to the mutual boundary.

**Figure 2:**
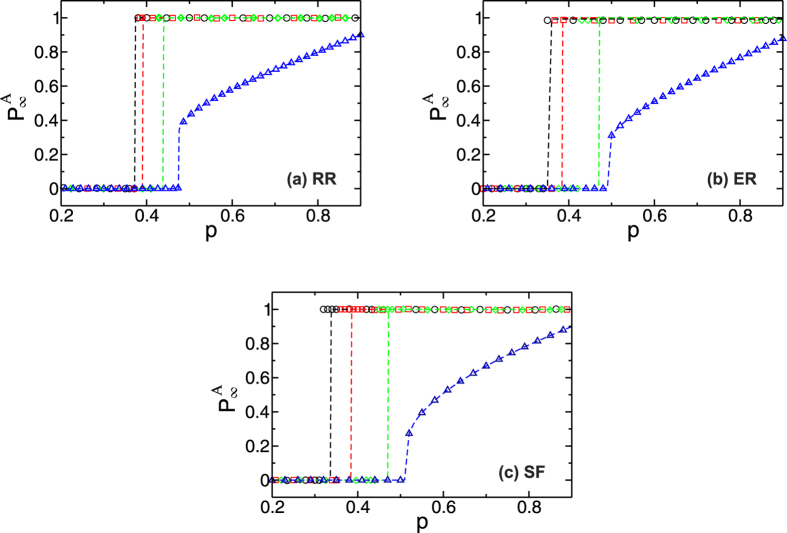
Fraction of nodes in the GC of network *A*, 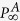, in the steady state of network *A* as a function of *p* for *N* = 10^6^ with *γ* > 0 (∇), *γ* = 0.1 (◇), *γ* = 0.5 (□) and *γ* = 1 (○) for (**a**) RR networks with *z* = 5, (**b**) ER networks with 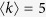 and (**c**) SF networks with *λ* = 3.0, with lower and maximal connectivity 3 and 1000 which correspond to 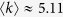. We include as a reference the no recovery case, *γ* = 0. The symbols correspond to the simulations and the dashed lines are the theoretical solutions of Eqs (2, 3, 4, 5, 6, 7). Simulations have been averaged over 1000 network realizations.

In Fig. 2 we show 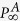 as a function of *p* in the steady state for three different systems of interdependent networks: two Random Regular (RR), two Erdös Rényi (ER) and two Scale Free (SF), characterized by a power law degree distribution with exponent *λ* = 3. In these plots we show simulation results (symbols) and theoretical results, presented below, (lines) for four values of *γ*. The case of *γ* = 0 is shown as a reference. The details of the simulations are presented in *Section: Methods*. We can see that the critical threshold *p*_*c*_ decreases when *γ* increases, and thus the networks become more robust. Note that in our restoring model a steady state is reached when the system is either fully functional or fully collapsed and that there is no intermediate state. In the Supplementary Information*: Analytical solutions for the fraction of nodes in the GCs* we show that in our model the only solutions for the fraction of nodes in the GC of both networks are either one or zero. This is in contrast to other models of cascading failures in interdependent networks without recovery^16^,^17^,^20^,^22^,^23^,^24^,^25^,^34^,^35^,^36^ where intermediate states exist. From Fig. 2 we can see that the agreement between the theory and the simulations is very good for all cases. We find that the case of coupled SF networks for *γ* > 0 is the one that presents the largest deviation in *p*_*c*_ compared with more homogeneous networks, such as the ER or the RR.

**Figure 3:**
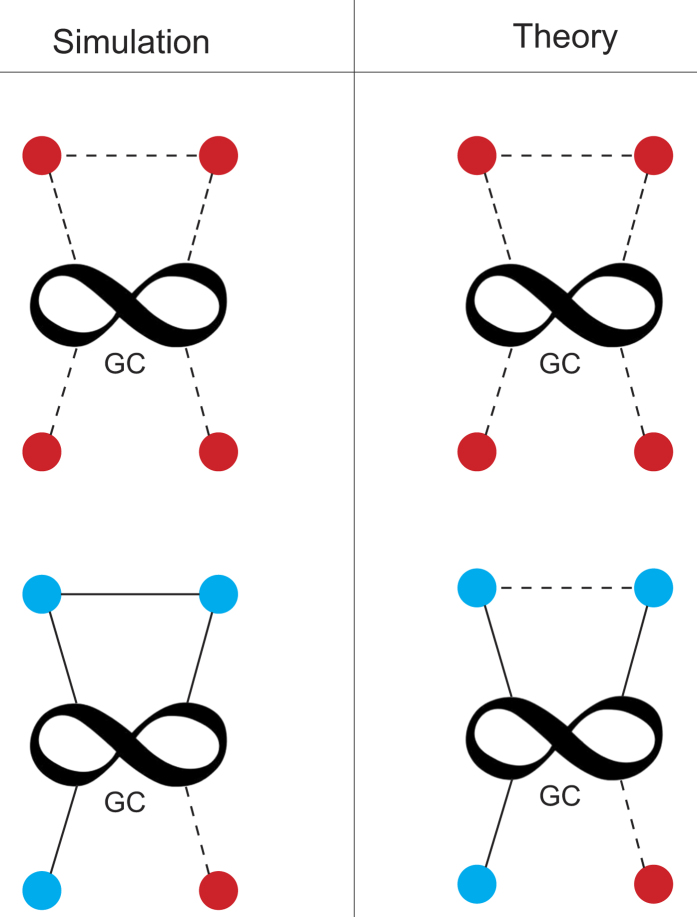
Schematic comparison of the rules of the recovery strategy between theory and simulations. In the left panel we show the rules for the simulations and in the right panel the rules for the theory. For simplicity we show only one of the coupled networks. The infinity symbol indicates the Giant Component (GC), red nodes are boundary failed nodes, while blue nodes are boundary recovered nodes. The dashed lines indicate inactive connections and the solid line reactivated connections. In the simulations a connection between two boundary recovered nodes is restored, which is not contemplated in the theory. In this case *γ* = 3/4, and boundary nodes have only one connection to the GC for simplicity.

To explain this deviation note that in our analytic approach we map node removal and repairing into random percolation. This means that in the theory all nodes have the same probability of failure and recovery. The repaired nodes are attached to the GC of their networks and cannot fail in the next step of the cascade of failures. It can be shown that the probability that a node belongs to the boundary increases with its degree (see Supplementary Information*: Excess Degree of the Boundary*), and this effect is more pronounced as the heterogeneity of the networks increases. In addition, recall that the simulation model reactivates broken connections between boundary restored nodes. This last feature of the model is illustrated in Fig. 3. These effects result in an increasing of the mean connectivity of the GC of each network. Thus the process of border recovery in the simulation generates a structure that is more resilient against failures than the structure in the theoretical approach, and therefore the critical thresholds of the theory are slightly higher from those of the simulations.

The relative deviation values of the simulation from the theory in the values of *p*_*c*_ are presented in Table S1 of Supplementary Information*: Deviations of the simulated threshold from the theoretical* .

**Figure 4:**
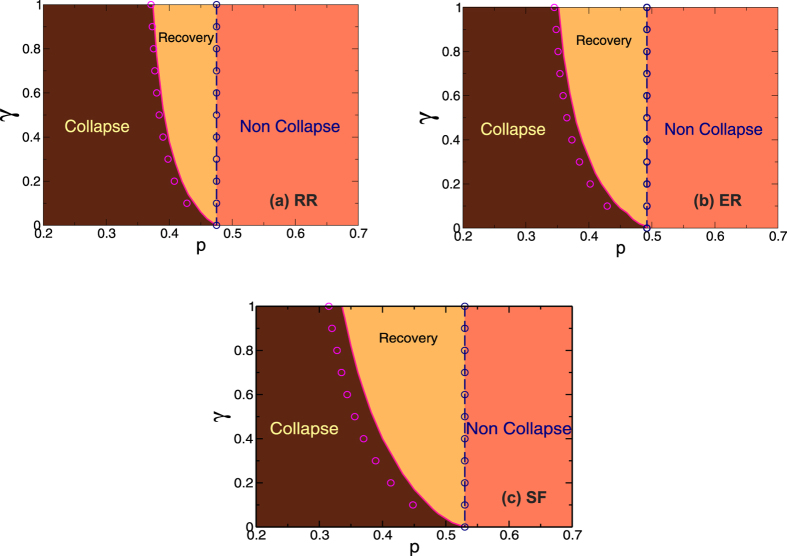
Phase diagrams in the γ − *p* plane obtained from the theoretical approach and simulations for (**a**) RR networks with *z* = 5, (**b**) ER networks with 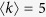 and (**c**) SF networks *λ* = 3 and 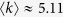. The symbols correspond to the simulations and the lines to the theory. The pink and magenta curves represent the values of *γ*_*c*_ as a function of *p*. The blue lines and symbols represent the values of *p*_*c*_ for *γ* = 0.

From the dependence of the order parameter on *γ* another useful measure can be obtained. If a system suffers a random initial removal of a fraction of (1 − *p*) of nodes, what is the minimum value of *γ* (probability of recovery) that prevents the collapse of the system? We denote this critical value *γ*_*c*_. In Fig. 4 we show the phase diagrams in the *γ* − *p* plane obtained from the simulations and theory for RR-RR with degree *z* = 5, ER-ER with 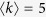, and SF networks with *λ* = 3 and minimum degree 3, which corresponds to 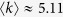. The symbols correspond to the simulations and the lines to the theory. We can see that the relation between *p* and *γ*_*c*_ is approximately the same for both curves, except for a small deviation in their left sides. Hence our analysis of the phase diagram is only based on the theory results. We can see that there are three well-defined regions, delimited by the solid curve, which represents the values of *γ*_*c*_ for each *p*, and by the dashed line, which indicates the value of *P*_*c*_ for *γ* = 0. The region located to the right of the dashed line is the non-collapsed region, since the system does not crash at any of these values of *p*. Note that the simulation points coincide exactly with this curve, as the theory has an excellent agreement with the simulations for *γ* = 0^20^. To the left of the dashed line and up to the solid line is the recovery phase in which there is always a minimum value of *γ*_*c*_ that prevents the collapse of the system. This region depends on 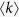 and shifts to the left (lower *p*) when the mean connectivity increases (see Fig. S1 in the Supplementary Information*: Phase Diagrams*), which means that the restoring process is needed more for lower values of 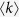. Finally, to the left of the solid (pink) curve is the collapsed phase in which the recovery process cannot prevent the complete breakdown of the system.

#### Number of Iteration Steps and Dynamics

**Figure 5:**
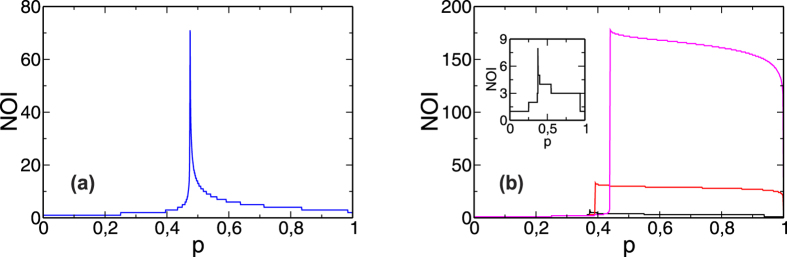
Theoretical Number of Iteration Steps (NOI) as a function of *p* for (**a**) *γ* = 0 (blue) and (**b**) *γ* = 1 (black), *γ* = 0.5 (red), *γ* = 0.1 (magenta) for RR networks with *z* = 5. The inset shows only the case *γ* = 1 for a better visualization.

An accurate approach that can also be used in structured networks—such as networks with communities, degree correlation, and clustering—is to extract the values of the critical threshold *p*_*c*_ for each *γ* from the number of iterations steps (NOI) in the cascading process, which exhibits a maximum at *p*_*c*_^35^. The NOI is the number of iterative cascade steps required for the system to reach the steady state. It is known that in a conventional cascade of failures without any process of recovery applied the NOI presents a very sharp peak at the critical threshold. This means that the system requires a long period of time to reach the steady state when *p* is close to *p*_*c*_, but when we move away from *p*_*c*_ the system reaches the steady state in a few steps. In Fig. 4 we show the theoretical values of the NOI for RR-RR networks for *γ* = 0, *γ* = 0.1, *γ* = 0.5, and *γ* = 1. We show only the results for RR coupled networks because for ER and SF networks they are qualitatively the same. Note that in Fig. 5(a) the NOI is clearly localized in the critical value and has a sharp peak when no strategy of recovery is applied. From Fig. 5(b) we can make two observations, (i) that the number of steps increases as *γ* decreases, and (ii) that the NOI does not present a sharp peak and has a flattened plateau form above *p*_*c*_. The first observation means that as the fraction of repaired nodes becomes larger the system requires fewer steps to reach the steady state at the critical point, and the second indicates that the required time for fully restoring the system is not strongly affected by the initial failure *p*.

**Figure 6:**
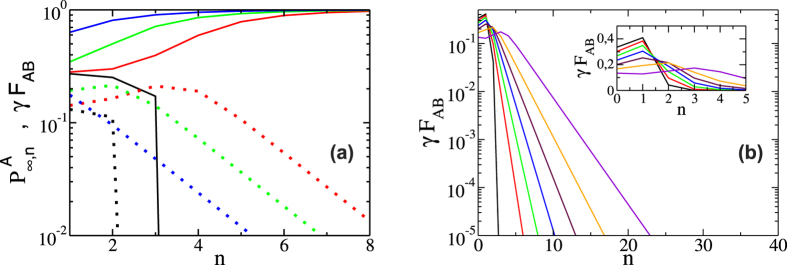
Log-Linear plot of (**a**) fraction of nodes in the GC of *A*, 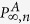 (solid lines) and the fraction of repaired nodes in the mutual boundary *γF*_*AB*_ (dot lines) as a function of *n* for *p* = 0.391 (black), *p* = 0.392 (red), *p* = 0.4 (green) and *p* = 0.48 (blue). The black full and dotted lines denote the value of *p* in the Collapse region. In red and green are the regions of Collapse-Recovery curves and in blue the Recovery region (**b**) fraction of repaired nodes in the mutual boundary *γF*_*AB*_ as a function of the iteration step *n* in the recovery region, with *p* = 0.4 from left to right *γ* = 1 to *γ* = 0.4 in intervals of 0.1. The inset shows the maximum of *γF*_*AB*_ located at the first steps of the process. The curves where obtained for RR networks with *z* = 5, from the theoretical approach.

To better understand this behavior we show in Fig. 6(a) in a log-linear scale for better visualization the temporal evolution of the order parameter and *γF*_*AB*_ in network *A* for RR networks with *z* = 5 and *γ* = 0.5 for different values of *p*, obtained from our theoretical approach. The red and blue lines separate the three regions of the phase diagram of Fig. 4, the collapsed, recovered and non-collapsed regions. Below the threshold in the collapsed phase the system becomes dysfunctional in a few steps, but just above the threshold there is a competition between the recovery process and the cascade of failures and thus the number of iteration steps greatly increases. Although the system is less damaged in the non-collapsed region than in the recovered region the amount of time the system needs to reach the steady state is approximately the same. This can be explained from the temporal behavior of the mutual restored boundary *γF*_*AB*_ shown in dashed lines in Fig. 6(a). As the number of steps increases and the system approaches full restoration, the mutual boundary exhibits a peak after which it decays exponentially. This shows that at each step of the cascade the number of nodes repaired becomes smaller. Thus because in our model the complete recovery of the system of networks requires that all single nodes be reactivated, the process of recovery always takes longer than in the collapsed region since it takes a long time to repair the few remaining non-functional nodes. This can be easily seen from Fig. 6(b) where we show a Log-Linear plot of *γF*_*AB*_ as a function of *n* for the same RR network as in Fig. 6(a) in the recovered region for different values of *γ* and *p* = 0.4. Note that the fraction of recovered nodes in the boundaries reaches a maximum in few steps, which shifts to the right as *γ* decreases. At the maximum the fraction of nodes in the GC is almost fully recovered as shown in blue in Fig. 6(a). After the maximum it decays exponentially in a characteristic time that increases as *γ* decreases, and as a consequence the dynamic of the system takes longer to fully recover.

## Discussion

We note that the dynamics of cascading failures when *γ* > 0 differ greatly from when *γ* > 0 (see Fig. 5 in *Section: Number of Iteration Steps and Dynamics*). The main difference is that when *p* > *p*_*c*_ and *γ* = 0^35^ the number of iteration steps (NOI) needed to reach the steady state decays sharply, but when *γ* > 0 it remains high. The reason for this difference is that the NOI only counts cascading failure steps when *γ* > 0, but when *γ* > 0 it also counts the steps of recovery to a fully functional system. The recovered region is characterized by a dynamic that is slower than in systems that undergo cascade of failures without recovering.

In summary, we have proposed and studied a recovery strategy to mitigate the breakdown of a system composed of two interdependent networks in the presence of cascading failures. The strategy consists of repairing with a probability *γ* every node that belongs to the mutual boundary of each GC. Our strategy yields the minimal probability, *γ*_*c*_, at which one can repair the components and prevent system collapse. We have solved the problem theoretically using random node percolation theory and have obtained a good agreement with the simulation results with small deviations close to the critical point. We believe that our model is an important contribution in developing a usable strategy for repairing damaged infrastructure systems, and that it also suggests future directions of research focused on recovery processes.

## Methods

For the simulations in RR-RR and ER-ER networks we use a system size of *N* = 10^6^, for SF-SF *N* = 5 10^6^ was used, and for the construction of the networks we use the Molloy-Reed Algorithm^37^ averaged over 1000 network realizations of the process. In the simulations, when *p* is close to the critical value *p*_*c*_, which depends on *γ*, the network collapses in some network configurations and is restored completely in others. For a fixed value of *p*, we consider the system fully recovered if the network is restored in more than 50% of the realizations and collapsed if it is restored in less than 50%. This statement is supported by our finding that this is a finite size effect, i.e., in the limit of infinite network size the system either collapses or is repaired completely for a given value of *p*.

To accurately evaluate the values of *p*_*c*_ as a function of *γ* using simulations, we compute the value of *p* at the peak of the number of iteration steps (NOI) needed to reach the steady state^35^ (see *Section: Number of Iteration Steps and Dynamics*).

As the process of measuring the peak in the NOI requires heavy data analysis, we compute the theoretical values of *γ*_*c*_ in the phase diagram as follows. For a fixed value of *p* the theoretical process is evaluated for varying values of *γ*. When the GCs drop to zero, we record the *γ* value to be *γ*_*c*_ = *γ*_*c*_(*p*). We find numerically for all studied cases that these values coincide with the ones obtained from the peak of the NOI.

## Additional Information

**How to cite this article**: Di Muro, M. A. *et al.* Recovery of Interdependent Networks. *Sci. Rep.* **6**, 22834; doi: 10.1038/srep22834 (2016).

## Supplementary Material

Supplementary Information
