# Corrigendum: Recovery of Interdependent Networks

**DOI:** 10.1038/srep46586

**Published:** 2017-04-26

**Authors:** M. A. Di Muro, C. E. La Rocca, H. E. Stanley, S. Havlin, L. A. Braunstein

Scientific Reports 6: Article number: 22834; 10.1038/srep22834 published online: 03 09 2016; updated: 04 26 2017.

The Supplementary Information file originally published with this Article omitted Reference 1, and a number of equations contained errors. Therefore,


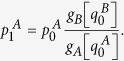


now reads:


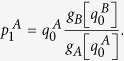



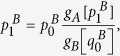


now reads:


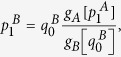



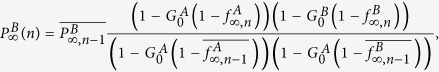


now reads:


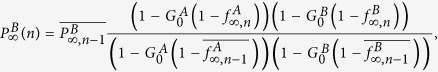


These errors have been corrected in the Supplementary Information that now accompanies the Article.

