# Supplementary material for: Recovery of Interdependent Networks
Source: Sci Rep. 2016 Mar 9;6:22834. doi: 10.1038/srep22834 (PMC4783785; doi:10.1038/srep22834)
Supplement: Supplementary Information [file srep22834-s1.pdf]

Recovery of Interdependent Networks

M. A. Di Muro,[*] C. E. La Rocca, H. E. Stanley, S. Havlin, and L. A. Braunstein

Supplementary Information

Theory

In this section we explain in detail how we generalize our process to include recovery. At the initial stage, $n = 0$, the fraction of nodes in the GC of network A is given by

$$P^A_{\infty,0} = g_A[p^A_0] = g_A[p] \ .$$

The failure next propagates to network B in which the fraction of remaining nodes is

$$p^B_0 = g_A[p] \ ,$$

and hence

$$P^B_{\infty,0} = g_B[p^B_0] = g_B[g_A[p]] \ .$$

After the initial failure moves from network A to B, the process of recovery begins. The nodes that are repaired are those that belong to the mutual boundary of both GCs. We calculate the fraction of nodes that are in the border of each GC to be

$$\begin{aligned} F^A_0 &= (1-p)\left(1 - G^A_0[1 - f^A_{\infty,0}]\right) \ , \\ F^B_0 &= (1 - g_B[g_A[p]])\left(1 - G^B_0[1 - f^B_{\infty,0}]\right) \ , \end{aligned} \quad \text{(S1)}$$

and the mutual boundary, given by

$$F^{AB}_0 = F^B_0 \frac{F^A_0}{1 - g_A[p]} \ ,$$

where $g_A[p]$ is the relative size of the GC in network A after the cascading failure, and $F^A_0/(1 - g_A[p])$ is the conditional probability that a node belongs to the boundary of the GC of network A ,given that it is interconnected though an interdependent link with a node that belongs to the boundary of the GC of network B.

---

[*]Electronic address: mdimuro@mdp.edu.ar

We next compute the new fraction of nodes that belong to the GC of each network

$$\overline{P^A_{\infty,0}} = g_A[p] + \gamma F_0^{AB} ,$$
$$\overline{P^B_{\infty,0}} = g_B[g_A[p]] + \gamma F_0^{AB} , \tag{S2}$$

and the fraction of functional nodes in each network after the recovery process by solving

$$g_A[q_0^A] = \overline{P^A_{\infty,0}} ,$$
$$g_B[q_0^B] = \overline{P^B_{\infty,0}} . \tag{S3}$$

We next compute the fraction of nodes remaining in network A in the next step of the cascade as in Ref. [**?**]

$$p_1^A = p_0^A \frac{g_B[q_0^B]}{g_A[q_0^A]} .$$

Hence the GC of A at $n = 1$ is given by

$$P^A_{\infty,1} = g_A[p_1^A] ,$$

and then the fraction of remaining nodes in B is

$$p_1^B = p_0^B \frac{g_A[p_1^B]}{g_B[q_0^B]} ,$$

and the fraction of nodes in its GC can we written as

$$P^B_{\infty,1} = g_B[p_1^B] .$$

Then the recovery process is applied again.

Analytical solutions for the fraction of nodes in the GC's

In this section we show that in the steady state when there are no isolated nodes before the initial failure the only possible values of the order parameter are 0 or 1, below and above the threshold without intermediate states.

Note that using Eqs. (1)–(7) in the main text we can write the temporal evolution of the order parameters as

$$P^A_{\infty,n} = \overline{P^B_{\infty,n-1}} \frac{(1 - G_0^A(1 - f^A_{\infty,n}))}{(1 - G_0^A(1 - \overline{f^A_{\infty,n-1}})} ,$$

$$P^B_\infty(n) = \overline{P^B_{\infty,n-1}} \frac{(1-G^A_0(1-f^A_{\infty,n}))(1-G^B_0(1-f^B_{\infty,n}))}{(1-G^A_0(1-\overline{f^A_{\infty,n-1}}))(1-G^A_0(1-\overline{f^B_{\infty,n-1}}))} ,$$

where $f^\alpha_{\infty,n}$ and $\overline{f^\alpha_{\infty,n}}$ with $\alpha = A, B$ satisfy the trascendental equations

$$f^A_{\infty,n} = \overline{P^B_{\infty,n-1}} \frac{(1-G^A_1(1-f^A_{\infty,n}))}{(1-G^A_0(1-\overline{f^A_{\infty,n-1}}))} ,$$

$$f^B_{\infty,n} = \overline{P^B_{\infty,n-1}} \frac{(1-G^A_0(1-f^A_{\infty,n}))(1-G^B_1(1-f^B_{\infty,n}))}{(1-G^A_0(1-\overline{f^A_{\infty,n-1}}))(1-G^B_0(1-\overline{f^B_{\infty,n-1}}))} ,$$

$$\overline{f^A_{\infty,n}} = \overline{P^A_{\infty,n}} \frac{(1-G^A_1(1-\overline{f^A_\infty, n}))}{(1-G^A_0(1-\overline{f^A_{\infty,n}}))} ,$$

$$\overline{f^B_{\infty,n}} = \overline{P^B_{\infty,n}} \frac{(1-G^B_1(1-\overline{f^B_{\infty,n}}))}{(1-G^B_0(1-\overline{f^B_{\infty,n}}))} .$$

and $\overline{P^\alpha_{\infty,n}} = P^\alpha_{\infty,n} + FAB_n$, with $\alpha = A, B$, where FAB_n is the shared boundary.

In the steady state at $n = n_s$, $P^A_{\infty,n_s} = P^B_{\infty,n_s}$. It is straightforward to show that $P^A_{\infty,n_s} = P^B_{\infty,n_s} = 0$ is a solution of the previous system of equations. For $P^B_{\infty,n_s} > 0$ after some algebra it can be shown that

$$\overline{f^A_{\infty,n_s-1}} = f^A_{\infty,n_s} ,$$

$$\overline{f^B_{\infty,n_s-1}} = f^B_{\infty,n_s} .$$

Using these equalities we find

$$P^B_{\infty,n_s} = \overline{P^B_{\infty,n_s-1}} .$$

On the other hand, it is clear that at the steady state $P^A_{\infty,n_s} = P^A_{\infty,n_{s+1}}$. Using this relation and the previous results we deduce

$$\overline{P^B_{\infty,n_s}} = P^B_{\infty,n_s} \ .$$

Recalling that $\overline{P^B_{\infty,n_s}} = P^B_{\infty,n_s} + \gamma\ FAB_{n_s}$, hence we have

$$\gamma\ FAB_{n_s} = 0 \ .$$

Thus for $\gamma > 0$ the shared boundary in the steady state must be zero. Note that the condition $FAB_{n_s} = 0$ is trivially satisfied when $P^\alpha_{\infty,n_s} = 0$. Moreover, it can be shown using L'Hôpital's rule that the condition is also fulfilled when $P^\alpha_{\infty,n_s} = P^\alpha_{\infty,-1}$, where $P^\alpha_{\infty,-1}$ is the original fraction of nodes in each GC before the initial failure. On one hand, if each initial GC equals the whole network then $P^\alpha_{\infty,n_s} = 1$. On the other hand, if before the initial random failure the probability of existence of isolated nodes is not equal to zero $(P(k=0) \gtrapprox 0)$, such as in ER networks, then the initial fraction of nodes in each GC is not the entire newtork and thus $P^\alpha_{\infty,n_s} \lessapprox 1$.

Deviations of the simulated threshold from the theoretical

The theoretical results adjust well to the simulation results, except for small deviations when $\gamma > 0$. Using the phase diagrams in Fig. 4 in the main text, we compute these deviations as relative errors between the theoretical and simulated values. The relative error is defined as

$$\epsilon_r = 1 - \frac{p^s_c}{p^t_c},$$

where p^t_c and p^s_c are the critical values obtained from theory and simulations, respectively. Note that $p^s_c \leq p^t_c$ as explained in the *Theoretical Approach* section in the main text. In Table S1 the relative deviations are listed for several values of γ and for the three types of network.

Note that the deviations do not exceed 3% for RR, 5% for ER, and 8% for SF. The numerical simulations give results that are very similar to those from theory, and we now explore the interesting features derived primarily from theory.

γ	RR-RR	ER-ER	SF-SF
0.0	0.0	0.0	0.0
0.1	0.027	0.036	0.053
0.2	0.028	0.042	0.066
0.3	0.017	0.030	0.074
0.4	0.020	0.045	0.075
0.5	0.020	0.032	0.075
0.6	0.023	0.030	0.073
0.7	0.028	0.027	0.072
0.8	0.016	0.025	0.063
0.9	0.011	0.022	0.0067
1.0	0.008	0.020	0.062

TABLE S1: Relative deviation ϵ_r of the critical theshold p_c for different values of γ for a system composed of two RR networks (second column) with $z = 5$, two ER with $\langle k \rangle = 5$ (third column) and two SF networks with $\langle k \rangle \approx 5.11$ (fourth column).

Excess Degree of the Boundary

We next explain why the nodes on the boundary of the GC have higher degrees than dysfunctional nodes that are not on the boundary. For simplicity we will drop the network indices A and B in the main magnitudes. The boundary of the GC is the set of nodes that have at least one connection with the GC. The equation that represents the relative fraction of nodes that belongs to the GC is

$$P_\infty = \sum_{k=k_{min}}^{k_{max}} \tilde{p} P(k)(1 - (1 - f_\infty)^k) \ , \tag{S4}$$

where f_∞ is the root of the self-consistent Eq. (1) in the main text, and $\tilde{p}$ is the fraction of remaining nodes before repairing process is initiated.

We can rearrange the coefficients of Eq. (S4) as $P(k)(\tilde{p} - \tilde{p}(1 - f_\infty)^k)$, where $\tilde{p}$ is the fraction of remaining nodes, and $\tilde{p}(1 - f_\infty)^k$ is the probability that a non-failed node does not belong to the GC. Since for $\tilde{p} < 1$, $f_\infty < 1$, the probability that a node belongs to a finite cluster after failure decreases with k. Hence it is more likely for a node to be part of the GC if its connectivity is fairly high. The fraction of nodes that belongs to the boundary is obtained by simply replacing in Eq. (S4) $\tilde{p}$ with $1 - \tilde{p}$

$$F = \sum_{k=k_{min}}^{k_{max}} (1-\tilde{p})P(k)(1-(1-f_\infty)^k) \ .$$

Rearranging the coefficients we have $P(k)((1-\tilde{p})-(1-\tilde{p})(1-f_\infty)^k$ where $P(k)((1-\tilde{p})$ is the probability that a node has failed and $(1-\tilde{p})(1-f_\infty)^k$ holds for the probability that a node has failed and is not connected to the GC. As explained above, this last term decreases as k increases. Hence the probability that a node belongs to the boundary of the GC increases with its degree k.

Phase Diagrams

In Fig. S1 we show the phase diagrams in the $\gamma - p$ plane obtained from theory for different values of $\langle k \rangle$ for RR, ER and SF networks. As indicated in the main text, the recovery regions determined by the critical values of γ_c for each p (solid line) and by the value of p_c for $\gamma = 0$ (dashed line) shift to the left (lower p) when the mean connectivity increases, indicating that the restoring process is more essential when the $\langle k \rangle$ values are lower.

Note that the recovery regions for the SF-SF networks are the broadest for the same value of $\langle k \rangle$, i.e., of the three types of network they have the widest range of p values in which this restoring strategy is effective. On the other hand, the RR-RR networks have the most narrow recovery region. This difference is because the SF-SF networks have the largest degree dispersion and in the RR-RR networks it is null. This corroborates that large heterogeneity implies high resilience

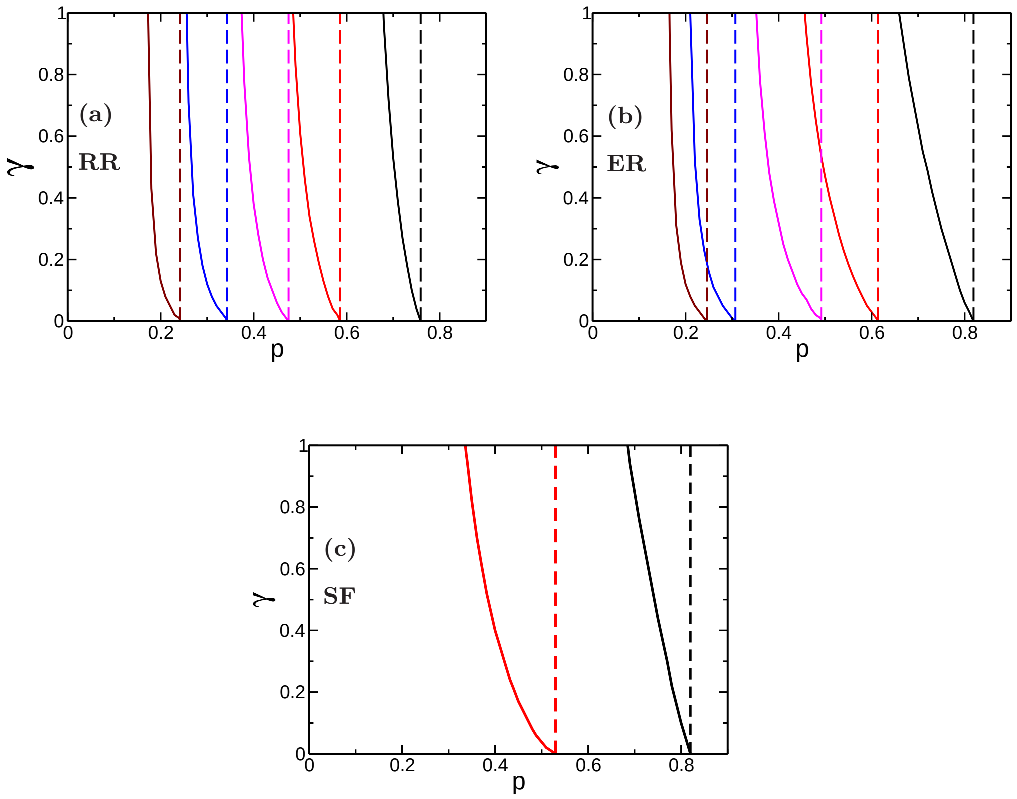

FIG. S1: (Color online). Phase diagram in the plane $\gamma - p$ for (a) RR networks with $z = 3$ (black), $z = 4$ (red), $z = 5$ (magenta), $z = 7$ (blue) and $z = 10$ (brown) (b) ER networks with $\langle k \rangle = 3$ (black), $\langle k \rangle = 4$ (red), $\langle k \rangle = 5$ (magenta), $\langle k \rangle = 7$ (blue) and $\langle k \rangle = 10$ (brown). For SF networks we used $\lambda = 3$ and minimum degrees 2 (black) and 3 (red). The recovery regions are enclosed by their respective curves. The curves were constructed from the theoretical values.
